# Establishment and characterization of an immortalized epicardial cell line

**DOI:** 10.1111/jcmm.16496

**Published:** 2021-04-06

**Authors:** Haobin Jiang, Shen Song, Jiacheng Li, Qianqian Yin, Shengshou Hu, Yu Nie

**Affiliations:** ^1^ State Key Laboratory of Cardiovascular Disease Fuwai Hospital National Center for Cardiovascular Disease Chinese Academy of Medical Sciences and Peking Union Medical College Beijing China; ^2^ Biodynamic Optical Imaging Center and Center for Reproductive Medicine College of Life Sciences Third Hospital Peking University Beijing China

**Keywords:** cell isolation, cell model, epicardium, immortalization, SV40‐LT

## Abstract

Recently, the increasing significance of the epicardium in cardiac development and regeneration is beginning to be recognized. However, because of the small proportion of primary epicardial cells and the limited cell culture time, further research on the mechanism of epicardial cells is hindered. Here, we transfected *simian virus 40 Large T (SV40‐LT)* into primary epicardial cells to establish an immortalized cell line, named EpiSV40. We further demonstrated that EpiSV40 can be easy to culture and has the proliferation, migration and differentiation capacities comparable to primary epicardial cells. EpiSV40 can serve as an ideal in vitro model for epicardial cell research, which will booster the study of the epicardium in cardiac development and heart regeneration.

## 1 INTRODUCTION

The epicardium is the outermost mesothelial cell layer covering the vertebrate heart, which plays an important role under physiological and pathological conditions.^1^ In embryonic heart development, the epicardium plays a crucial role as the source of several mitogenic signals that provide trophic support for myocardial growth and generates epicardial‐derived cells (EPDCs) through epithelial to mesenchymal transition (EMT) to give rise to various cardiac lineages, including fibroblasts, vascular smooth muscle cells, pericytes, endothelial cells, with controversial contributions to cardiomyocytes.^1^, ^2^ After birth, the epicardial cells enter a quiescent cell state but can be activated immediately in response to heart damage, which secretes a variety of cytokines and undergoes cell differentiation to promote the repair of the heart.^3^, ^4^, ^5^, ^6^, ^7^, ^8^ Presently, the epicardial cells have been used as an effective tool to advance cardiovascular regenerative medicine, including cell transplantation therapy and tissue‐engineering approaches,^9^, ^10^, ^11^ while the underlying molecular mechanisms of epicardial cells are still largely unknown.

In vitro cellular experiment is an important strategy for investigating the molecular mechanisms of epicardium function, but the primary culture of epicardial cells from embryonic mouse heart faces challenges. The epicardial cell isolation is a laborious and time‐consuming process, which required a 14‐day lead time for pregnant mice and microscopic manipulation of embryos.^12^ Besides, the amount of epicardial cells is limited and cannot expand in vitro.^13^, ^14^ Hence, it is necessary to establish a cell line that could maintain the primary epicardial cell characterization for mechanism research.

Introgression of exogenous genes like Epstein‐Barr virus,^15^ simian virus 40 Large T ^16^ and telomerase reverse transcriptase is commonly used for immortalization of primary cells.^17^ The simian virus SV40, a polyomavirus of rhesus macaques, was identified in 1960, which encodes two proteins, large T antigen and small t antigen.^18^, ^19^ The large T antigen of SV40 (*SV40‐LT*) has been widely used for primary cell immortalization by regulating the p53‐mediated cell cycle.^16^ *SV40‐LT*‐mediated cell immortalization has been applied to melanocyte cells, glomerular podocytes, atrial myocytes, ventral mesencephalic neuronal progenitor cells, dental mesenchymal cells and retinal microglial cells.^20^, ^21^, ^22^, ^23^, ^24^, ^25^

In this study, we transfected the *SV40‐LT* into the primary epicardial cells isolated from embryonic mouse hearts and established an immortal cell line, named EpiSV40. Characterization of EpiSV40 revealed similarities to primary epicardial cells concerning cell morphology and capacity of cell proliferation, migration and differentiation. With EpiSV40, researchers can perform in vitro epicardial cell mechanism study conveniently, saving costs both in terms of time and effort. The established EpiSV40 serves as a model for further study of the epicardium in cardiac development and heart repair.

## 2 MATERIALS AND METHODS

### 2.1 Mice

All experiments involving animals were conducted following the Guide for the Use and Care of Laboratory Animals. All animal protocols were approved by the Institutional Animal Care and Use Committee (IACUC), Fuwai Hospital, Chinese Academy of Medical Sciences. The wild‐type C57BL/6J adult mice were obtained from SPF (Beijing) Biotechnology Co. Ltd. for experiments. The *WT1^GFPCre/+^* and *Rosa26‐RFP ^f/+^* mice lines were kindly gifted by Dr Bin Zhou, Chinese Academy of Sciences.^26^ For timed pregnancies, mid‐day of vaginal plug was considered as embryonic day 0.5 (E0.5).

### 2.2 Immunohistochemistry

Immunohistochemistry was performed as described previously.^27^ Briefly, embryos were dissected in PBS and fixed in 4% paraformaldehyde overnight at 4°C. Embryos were washed with PBS, dehydrated in ethanol and embedded in paraffin. Sections of 5 μm in thickness were deparaffinized in xylene and rehydrated through the graded ethanol series (100%, 95%, 75% and 50%), then stained for immunohistochemical detection. Immunohistochemical detection was performed on paraffin sections of paraformaldehyde‐fixed hearts. Heat‐mediated antigen retrieval using EDTA solution was applied to the sections. For immunohistochemical detection of cell explants, cell explants washed in PBS and fixed in 4% paraformaldehyde for 10 minutes at 4°C. Then tissue sections or cell explant chambers were blocked in 5% donkey serum (Invitrogen) with 0.3% Triton X‐100 (Sigma) and incubated with primary antibodies overnight at 4°C. The sample was washed 5 minutes 5 times with PBS, and thereafter, the samples were incubated with secondary antibodies which were conjugated to Alexa Fluor 488/594/647 (Invitrogen) for 1 hour at 37°C. Fluorescence was observed under a confocal laser scanning microscope (ZEISS LSM800). Primary antibodies used for immunohistochemistry were anti‐Tnnt2 mouse monoclonal (Abcam, Cat. no. ab8295), anti‐WT1 rabbit polyclonal (Abcam, Cat. no. ab89901), anti‐RALDH2 rabbit polyclonal (Abcam, Cat. no. ab96060), anti‐GFP goat polyclonal (Novus, Cat. no. NB100‐1770SS), anti‐SV40 T Ag (Pab 101) mouse monoclonal (SANTA CRUZ BIOTECHNOLOGY, Cat. no. sc‐147), anti‐ phospho‐histone H3 (pH3) rabbit polyclonal (Millipore, Cat. no. 06‐570), anti‐Ki67 rat monoclonal (Invitrogen, Cat. no. 14‐5698‐82), anti‐α‐SMA rabbit polyclonal (Abcam, Cat. no. ab5694) and anti‐β‐catenin rabbit monoclonal (Abcam, Cat. no. ab32572).

### 2.3 Isolation and culture of primary epicardial cells

Hearts from E11.5 embryos are isolated in a sterile environment and placed in a 24‐well plate which is coated with 1% gelatin. Slowly add 250ul DMEM containing 10% FBS along the wall of the 24‐well plate until the liquid just covers half of the heart (too much medium will cause the heart to float away from the centre because of the liquid surface pressure, while too little medium will cause the migrated cell death because of the loss of the medium supply). Hearts are incubated for 8 hours in a CO_2_ incubator at 37°C. Then another 250 μl medium is added to the well and continue to culture for 24 hours. After that, it can be seen that the primary epicardial cells migrated onto the well in a circular shape around the heart. Then hearts are sucked from the well with a 200ul yellow pipette tip, the medium was changed every 48 hours thereafter.

### 2.4 EdU incorporation

EdU was used to label primary epicardial cells and EpiSV40 undergoing mitosis in vitro. Cells grown on gelatin‐coated chamber‐slides were treated with 10 μM EdU for 2 hours in culture medium and subjected to EdU immunostaining. EdU incorporation was detected using BeyoClick™ EdU Cell Proliferation Kit with Alexa Fluor 488 (Beyotime catalog no. C0071S).

### 2.5 Quantitative reverse transcription‐polymerase chain reaction (qRT‐PCR)

Total RNA was isolated from cells using TRIzol (Life Technologies, Cat. no. 15596‐018). The PrimeScript RT Master Mix was used to convert RNA into cDNA. qRT‐PCR for the analysis of expression of different genes (Table 1) was performed in triplicate using the SYBR Green qPCR Master Mix (Applied Biosystems) in a QuantStudio 5 Real‐Time PCR Systems (Applied Biosystems). The results were analysed using GraphPad Prism and differences among groups were analysed using Student's *t* test. Significance levels are indicated by Prism Software as follows: *P < 0.05, **P < 0.01, ***P < 0.001.

**TABLE 1:** The primer sequences for qRT‐PCR

Gene	Primer sequences
*Wt1*	F: 5′‐ GAGAGCCAGCCTACCATCC ‐ 3′
	R: 5′‐ GGGTCCTCGTGTTTGAAGGAA ‐ 3′
*Raldh2*	F: 5′‐ GGCACTGTGTGGATCAACTG ‐ 3′
	R: 5′‐ TCACTTCTGTGTACGCCTGC ‐ 3
*Krt14*	F: 5′‐ AGCGGCAAGAGTGAGATTTCT ‐ 3′
	R: 5′‐ CCTCCAGGTTATTCTCCAGGG ‐ 3′
*Tcf21*	F: 5′‐ CATTCACCCAGTCAACCTGA ‐ 3′
	R: 5′‐ CCACTTCCTTCAGGTCATTCTC ‐ 3′
*Tjp‐1*	F: 5′‐ GCCGCTAAGAGCACAGCAA ‐ 3′
	R: 5′‐ TCCCCACTCTGAAAATGAGGA ‐ 3′
*Snail2*	F: 5′‐ TGGTCAAGAAACATTTCAACGC ‐ 3′C
	R: 5′‐ GGTGAGGATCTCTGGTTTTGGTA ‐ 3′
*Twist1*	F: 5′‐ GGACAAGCTGAGCAAGATTCA ‐ 3′
	R: 5′‐ CGGAGAAGGCGTAGCTGAG ‐ 3′
*Cdh2*	F: 5′‐ AGCGCAGTCTTACCGAAGG ‐ 3′
	R: 5′‐ TCGCTGCTTTCATACTGAACTTT ‐ 3′
*Nkx2.5*	F: 5′‐ GACGTAGCCTGGTGTCTCG ‐ 3′
	R: 5′‐ GTGTGGAATCCGTCGAAAGT ‐ 3
*Tnnt2*	F: 5′‐ ATTCGCAATGAGCGGGAGAA ‐ 3′
	R: 5′‐ ACCCTCCAAAGTGCATCATGT‐ 3
*SV40‐LT*	F: 5′‐ AAGTTTAATGTGGCTATGGG‐ 3′
	R: 5′‐ ACTGTGAATCAATGCCTGTT‐ 3
*Tbx18*	F: 5′‐ GTACCTGGCTTGGCACGAC‐ 3′
	R: 5′‐ GCATTGCTGGAAACATGCG‐ 3
*Snail1*	F: 5′‐ CACACGCTGCCTTGTGTCT‐ 3′
	R: 5′‐ GGTCAGCAAAAGCACGGTT‐ 3
*Acta2*	F: 5′‐ GTCCCAGACATCAGGGAGTAA‐ 3′
	R: 5′‐ TCGGATACTTCAGCGTCAGGA‐ 3
*Cnn1*	F: 5′‐ TCTGCACATTTTAACCGAGGTC‐ 3′
	R: 5′‐ GCCAGCTTGTTCTTTACTTCAGC‐ 3
*Tagln*	F: 5′‐ CAACAAGGGTCCATCCTACGG‐ 3′
	R: 5′‐ ATCTGGGCGGCCTACATCA‐ 3
*Actb*	F: 5′‐ GGTACCACCATGTACCCAGG‐ 3′
	R: 5′‐ AAAACGCAGCTCAGTAACAGTC‐ 3

### 2.6 Karyotype analysis

The karyotype analysis was performed using standard G‐banding techniques. Briefly, EpiSV40 cells cultured in a T25 flask were incubated with 100 ng/ml colcemid at 37°C for 5 hours, followed by dissociation with trypsin/EDTA and centrifugation at 1000 rpm for 10 min. Cells were re‐suspended in 0.05 mol/L potassium chloride hypotonic solution and incubated for 20 minutes. Following centrifugations, the cells were re‐suspended in fixative and aspirated on the glass slide, followed by staining with Giemsa solution.

### 2.7 Western blot analysis

Cultured cells were trypsinized and incubated in RIPA lysis buffer with 1 mM phenylmethylsulphonyl fluoride (PMSF) (Beyotime Institute of Biotechnology, Beijing, China) at 4°C for 40 minutes. The protein samples were mixed with 4 × SDS loading buffer and 10 × SDS loading buffer for 10 minutes at 70°C. Subsequently, 10 μg of total protein was loaded onto 10% sodium dodecyl sulphate‐polyacrylamide gel electrophoresis and transferred to a nitrocellulose membrane. After blocking the non‐specific background staining, the membranes were incubated at 4°C overnight with the primary antibody: anti‐SV40 T Ag (1:1000). The membranes were washed with Tris‐buffered saline/0.1% Tween 20 (TBST) and incubated with secondary antibodies for 1 hour at room temperature. Signals were detected using Pierce™ ECL Western Blot Substrate (Thermo Fisher Scientific).

### 2.8 Droplet digital PCR analysis

For droplet digital PCR (ddPCR) sample preparation, genomic DNA was extracted using Wizard Genomic DNA Purification Kit (Promega) according to the manufacturers’ instructions. DNA concentrations were determined using Qubit® 3.0 Fluorometer (Invitrogen) and Qubit dsDNA assay kit (Invitrogen, Q32852).

ddPCR was performed on a Bio‐Rad platform, using a QX200™ Droplet Digital™ PCR System (Bio‐Rad), a T100 Thermal Cycler (Bio‐Rad) and a PX1 PCR Plate Sealer (Bio‐Rad) with the following reagents: ddPCR Supermix for Probes (No dUTP) (1 863 024, Bio‐Rad), Droplet Reader Oil (Bio‐Rad, 1 863 004), Droplet Digital™ PCR Consumables (Bio‐Rad, 1 864 007) and ddPCR™ 96‐Well PCR Plates (Bio‐Rad, 12 001 925). The probes and primers were designed following Bio‐Rad's guidelines for ddPCR assays as followed:

*β‐actin*‐F: 5’‐ GGCTGTATTCCCCTCCATCG‐3’,
*β‐actin*‐R: 5’‐CGATCCCCAAGAAAACCCCA‐3’,
*β‐actin*‐P: FAM‐5’‐ CCCTAGGCACCAGGTAAGTGACC‐3’,
*SV40‐LT*‐F: 5’‐TCAGGGCATGAAACAGGCAT‐3’,
*SV40‐LT*‐R: 5’‐ GGGAGGTGTGGGAGGTTTT‐3’,
*SV40‐LT*‐P: HEX‐5’‐ACAGTCCCAAGGCTCATTTCAGGCCCC‐3’.

Reactions were performed with the following cycles: 10 minutes at 95°C, 40 cycles of 94°C for 30 s and annealing temperature for 60 s, and a final cycle of 10 minutes at 98°C. Following PCR, the droplets were read on the QX200 (Bio‐Rad), set for detecting absolute levels of FAM/HEX probe fluorophores.

### 2.9 Scratch migration assay

Cells were plated in 48‐well plates and cultured to confluency. Then, primary epicardial cells or EpiSV40 was serum‐starved and scraped with a 10 μL pipette tip (0 hour), washed with PBS and cultured with serum‐free DMEM supplemented with PBS or TGF‐β1 (R&D Systems, 10 ng/mL) for 48 hours. Images were taken at the indicated time points.

#### 2.9.1 Cell differentiation assay

Cells grown on gelatin‐coated chamber‐slides were treated with PBS or TGF‐β1 (R&D Systems, 10 ng/mL) for 72 hours in the culture medium and subjected to α‐SMA and β‐catenin immunostaining.

#### 2.9.2 Virus packaging and infection

Lentivirus packaging and cell infection were performed as described previously.^16^ Briefly, the pLVX‐IRES‐Puro‐SV40LT lentiviral expression vector was cotransfected with psPAX2 (Addgene, #12260) and pMD2.G (Addgene, #12259) packaging plasmids into HEK293T cells using Hieff Trans liposomal transfection reagent following the manufacturer's protocol (Yeasen). Every 24 hours for two times, the virus‐containing supernatant was collected and replaced with a fresh medium. The medium was cleared of cell debris by centrifugation at 1000 g for 5 minutes and used for infecting epicardial cells. The infected cells were selected with puromycin. The schematic diagram of plasmid pLVX‐IRES‐Puro‐SV40LT showed in Figure S1A and the detailed sequence information was added in Table S1.

### 2.10 FACS analysis

FACS was performed as described previously.^28^ In brief, embryonic mouse hearts from *WT1^GFPCre/+^* mice at E12.5 were dissected and dissociated into single cells using the Neonatal Heart Dissociation Kit (Miltenyi Bio‐Tech) with a heated shaking block at 37°C and 1000 rpm for 15 minutes. Cells suspension were then centrifuged at 300 g for 5 min and re‐suspended in DMEM with 1% BSA and kept on ice. FACS sorting of GFP populations was carried out by gating against a GFP‐negative control littermate. Samples were analysed by flow cytometry (BD FACS Arial II, BD bioscience), and the data processing was performed using FlowJo V.10 (Tree Star, Inc).

### 2.11 Statistical analysis

All results were analysed using GraphPad Prism (version 7.0, GraphPad Software). All data were expressed as the mean ± standard error of the mean (SEM). Differences between groups were evaluated using an unpaired Student's t test. Differences were considered significant when the *P*‐value was < 0.05.

## 3 RESULTS

### 3.1 Epicardium constitutes a low fraction of cardiac cells

**FIGURE 1:**
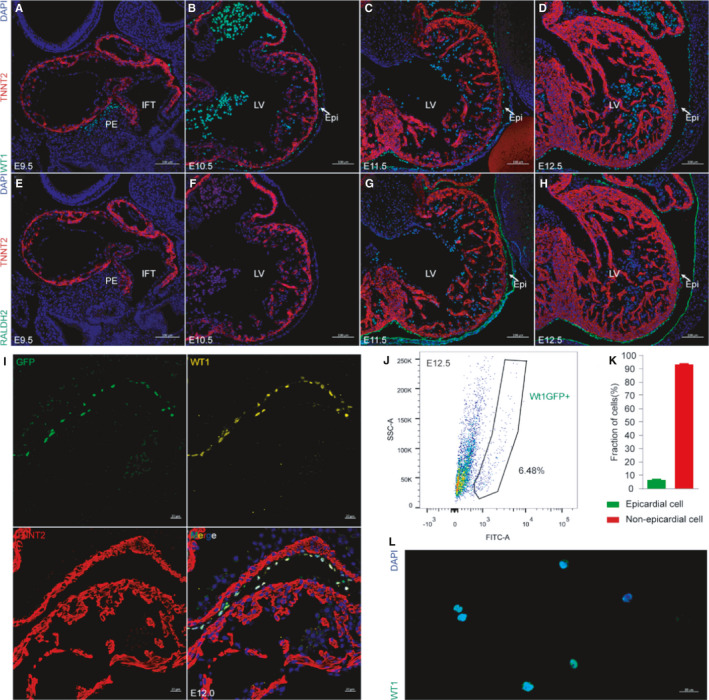
Epicardium constitutes a low proportion of cardiac cells. A‐H, Immunohistochemistry for WT1 and RALDH2 was performed on E9.5‐E12.5 mouse heart sections. TNNT2 was used to visualize cardiomyocytes (red) and DAPI was used to visualize nuclei (blue). PE, proepicardium. IFT, inflow tract. LV, left ventricle. Scale bar, 100 µm. I, Immunohistochemistry for GFP (green), WT1 (yellow), TNNT2 (red) and DAPI (blue) was performed on E12.0 *WT1^GFPCre/+^* mouse heart sections. Scale bar, 20 µm. J, Proportion of epicardial cells as analysed by flow cytometry‐based on their GFP expression. K, Fraction of epicardial cells of E12.5 *WT1^GFPCre/+^* mouse heart (n = 6 per group). L, Cell immunostaining for WT1 (green) and DAPI (blue) after FACS. Scale bar, 20 µm

We performed two typical markers of epicardium (WT1 and RALDH2) immunostaining on embryonic hearts from E9.5 to E12.5. At E9.5, WT1 expression was noted in the proepicardium (PE), which was a grape‐like cell protrusion from the venous pole of the developing heart tube, while Raldh2 was not expressed in epicardial cells before E10.5 (Figure 1A, B, E, F). After E11.5, the epicardium formed a thin cell monolayer covering the growing myocardium (Figure 1C, D, G, H). A recently reported single‐cell sequencing study has shown epicardial cells account for no more than 10% of all heart cells during the development.^29^ The *WT1^GFPCre/+^* mice line is a powerful reporter line to label the WT1‐positive epicardial cells with GFP expression (Figure 1I). We confirmed the low proportion of epicardial cells by Fluorescence‐activated Cell Sorting (FACS) analyses of E12.5 embryonic hearts from *WT1^GFPCre/+^* mice (Figure 1J, K). The immunofluorescence staining of WT1 further proved that the cells obtained from FACS were highly purified epicardial cells (Figure 1L). Taken together, these data demonstrated that the complete epicardial layer was formed after E11.5 which is suitable for cell culture and epicardial cells constitute a modest fraction of the foetal cardiac cells.

### 3.2 Serial passage of primary epicardial cells was limited

**FIGURE 2:**
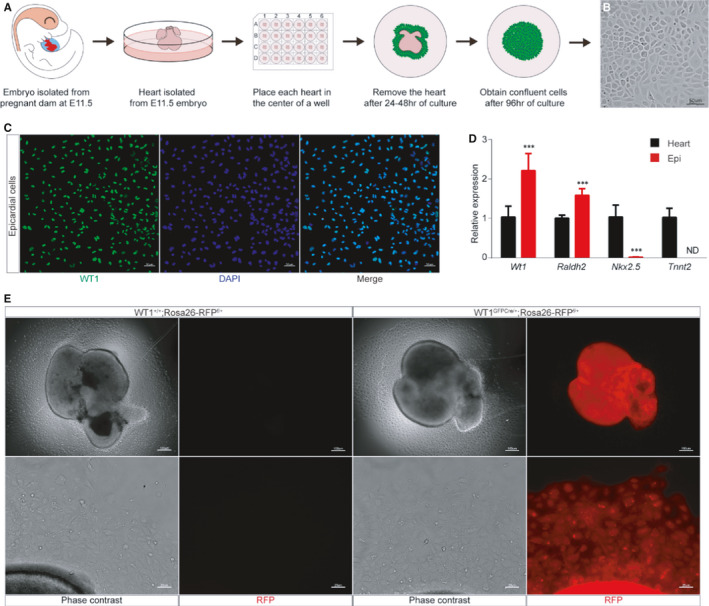
Primary epicardial cells isolation and evaluation of cell purity. A, A schematic outline of primary epicardial cells isolation workflow. B, Bright‐field images show cell morphology of primary epicardial cells. Scale bar, 100 µm. C, Immunohistochemistry for WT1 (green) and DAPI (blue) on primary epicardial cells. Scale bar, 50 µm. D, qRT‐PCR analysis of epicardial markers (*Wt1* and *Raldh2*) compared to myocardial gene expression (*Nkx2‐5* and *Tnnt2*) in corresponding epicardium‐depleted E11.5 hearts (n = 4 per group). Epi, primary epicardial cells. ND, not determined, ^∗^ *P* < 0.05, ^∗∗^ *P* < 0.01, and ^∗∗∗^ *P* < 0.001. E, Wholemount image of the heart of E11.5 *WT1^+/+^*; *Rosa26‐RFP ^f/+^* and *WT1^GFPCre/+^*; *Rosa26‐RFP ^f/+^* mouse and fluorescence images of RFP expression of migrated primary epicardial cells. Upper scale bar, 100 µm; Lower scale bar, 25 µm

Tissue attachment was adopted to separate highly purified primary epicardial cells from E11.5 mouse hearts. The schematic representation of the cell isolation strategy is shown in Figure 2A. The isolated epicardial cells showed typical epithelial cell characteristics of cobblestone morphology (Figure 2B). Immunofluorescence staining showed that these cells expressed WT1 (Figure 2C). Expression analysis by qRT‐PCR revealed robust expression of epicardial marker genes (*Wt1* and *Raldh2*) and low expression of myocardial genes (*Nkx2‐5* and *Tnnt2*) (Figure 2D). To further determined the authenticity of the acquired epicardial cells, we crossed the *WT1^GFPCre/+^* mice line with *Rosa26‐RFP ^f/+^* mice line to obtain the WT1‐Cre‐reporter mice *WT1^GFPCre/+^*; *Rosa26‐RFP ^f/+^*. The wholemount picture showed *WT1^GFPCre/+^*; *Rosa26‐RFP ^f/+^* heart had a robust expression of RFP, indicating that WT1–Cre labels epicardial cells efficiently. The fluorescence images showed all the migrating cells were labelled by RFP (Figure 2E). Taken together, these results revealed that the primary epicardial cells of high purity were successfully isolated by the tissue attachment method.

**FIGURE 3:**
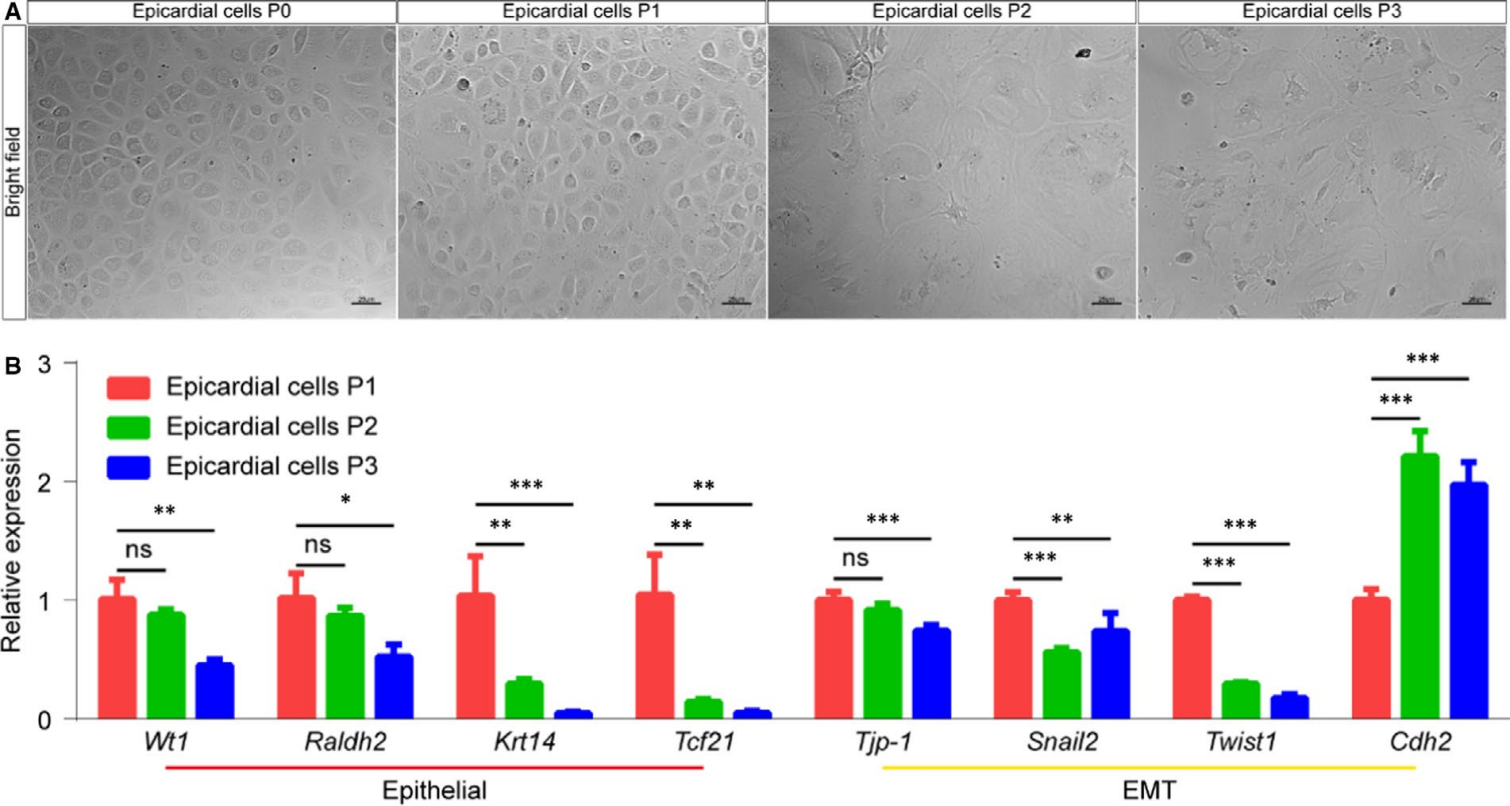
Evaluation of cell morphology and gene expression over passaging of primary epicardial cells. A, Bright‐field images show primary epicardial cell morphology. Scale bar, 25 µm. B, Fold changes in gene expression (n = 4 per group). EMT, epithelial to mesenchymal transition. NS, not significant, ^∗^ *P* < 0.05, ^∗∗^ *P* < 0.01 and ^∗∗∗^ *P* < 0.001

To expand the epicardial cell amount, we continuously passaged 3 generations from P0 to P3. We found that P0 and P1 were typical cobblestone‐like cells. However, the shape of the cells became large and flattened at P2 and P3 (Figure 3A), indicating those cells lost characteristics of the epicardial cell. The markers of the epicardial cell, including epithelial and EMT‐related genes, were detected by qRT‐PCR. We found that the expression of those marker genes was dramatically fluctuated over passaging (Figure 3B). Taken together, our results demonstrated that the serial passage of the primary epicardial cells in vitro remained limited. An immortalized epicardial cell lines are thus in high demand.

### 3.3 Establishment of an immortal epicardial cell line EpiSV40

**FIGURE 4:**
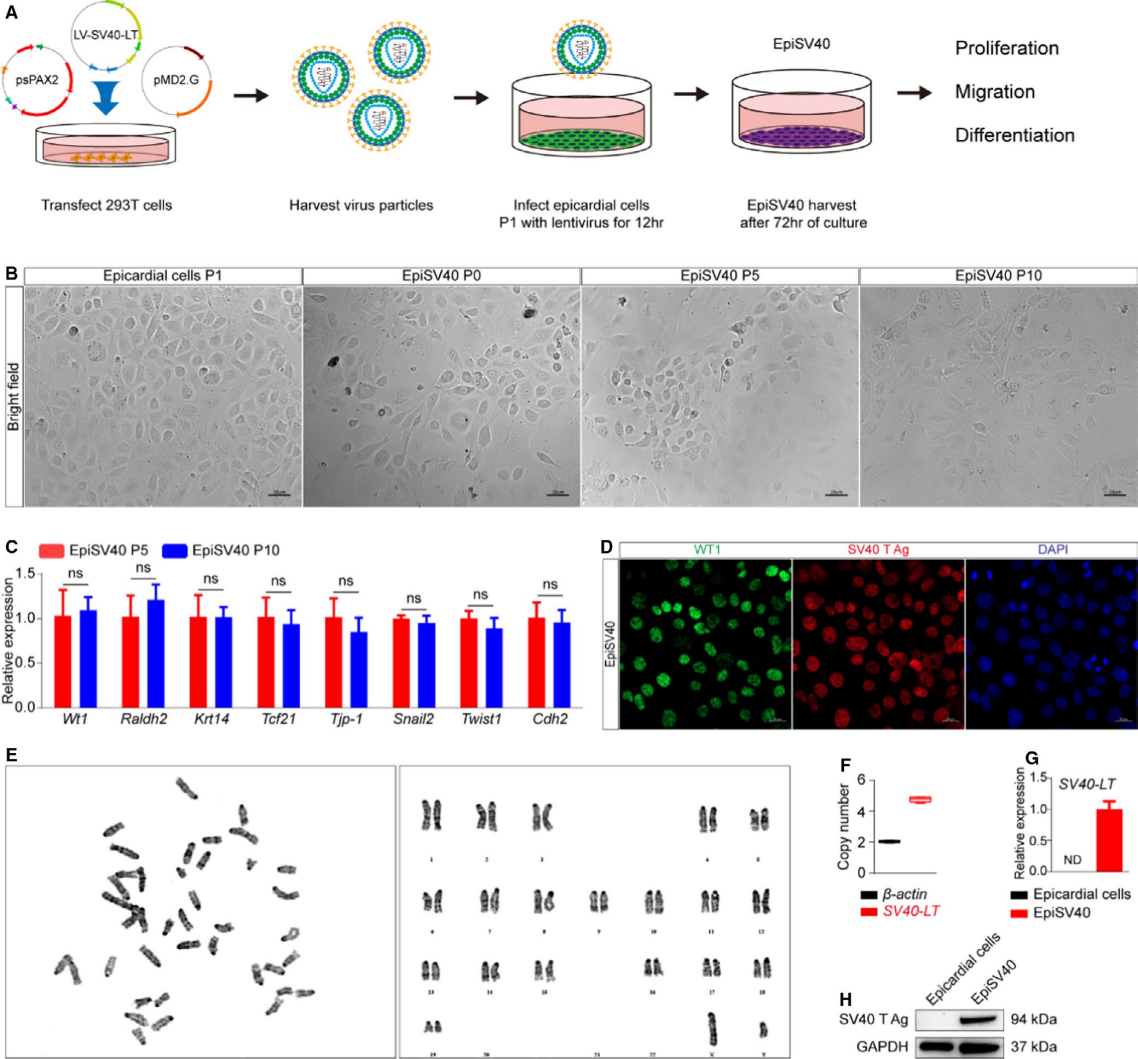
Evaluation of cell morphology and gene expression of EpiSV40. A, Schematic outline of the EpiSV40 preparation workflow. B, Bright‐field images show cell morphology of different passages of EpiSV40 and the primary epicardial cells P1. Scale bar, 25 µm. C, Fold changes in gene expression (n = 4 per group). EMT, epithelial to mesenchymal transition. NS, not significant, ^∗^ *P* < 0.05, ^∗∗^ *P* < 0.01 and ^∗∗∗^ *P* < 0.001. D, Immunohistochemistry for WT1 (green), SV40 T Ag (red) and DAPI (blue) was performed on EpiSV40. Scale bar, 20 µm. E, Karyotype analysis of EpiSV40 shows normal chromosome architecture. F, ddPCR analysis of copy number of *β‐actin* and *SV40‐LT* gene of EpiSV40 (n = 5 per group). G, Fold changes in gene expression of *SV40‐LT* (n = 4 per group). ND, not determined. H, Representative Western blot analysis of expression of SV40 T Ag

To establish an immortal epicardial cell model, we transfected *SV40‐LT* into the primary epicardial cells P1 separated from E11.5 mouse hearts and obtained a cell line named EpiSV40 (Figure 4A). Immunostaining, qPCR and Western blot analysis showed a robust expression of SV40 T antigen in EpiSV40, which confirmed the successful infection (Figure 4D,G‐H). Karyotype analysis of EpiSV40 was performed, showing 40 chromosomes (20 pairs), consistent with normal mouse chromosomes (Figure 4E). We further quantitatively established that on average EpiSV40 had 4.69 copies of the SV40‐LT gene in the genome by ddPCR assay (Figure 4F).

**FIGURE 5:**
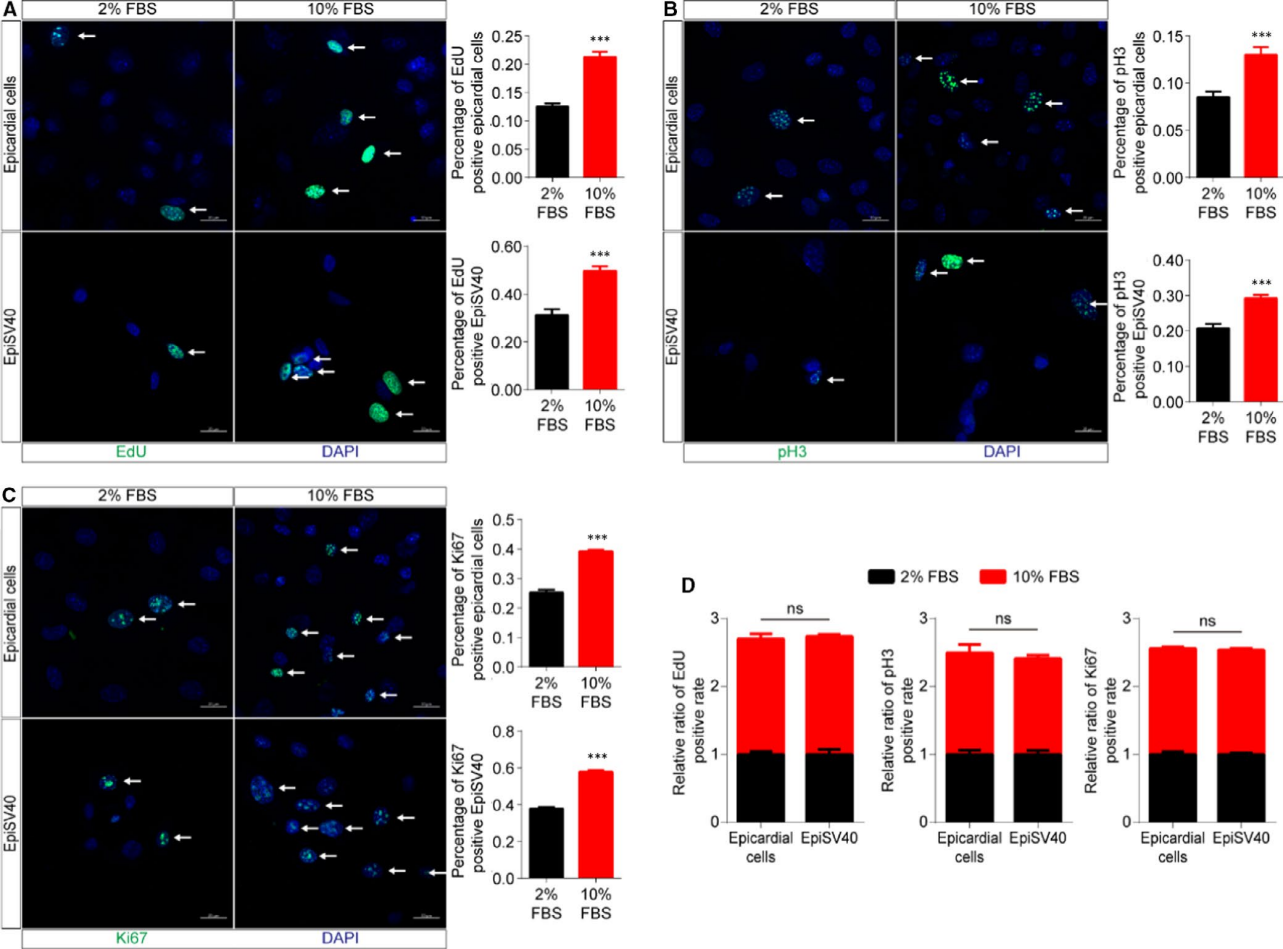
Proliferative capacity of primary epicardial cells and EpiSV40. A, Proliferating cells were detected via EdU (green) and DAPI (blue). Scale bar, 25 µm. B, Proliferating cells were detected via pH3 (green) and DAPI (blue). Scale bar, 25 µm. C, Proliferating cells were detected via Ki67 (green) and DAPI (blue). Scale bar, 25 µm. D, Relative ratio of proliferative rate via EdU, pH3 and Ki67 (n = 4 per group). NS, not significant, ^∗^ *P* < 0.05, ^∗∗^ *P* < 0.01 and ^∗∗∗^ *P* < 0.001

After ten passages, EpiSV40 still maintained typical cobblestone‐like morphology like primary epicardial cells P1 (Figure 4B). The expression of epicardial cell marker genes was not changed over passaging (Figure 4C). Compared to primary epicardial cells, the expression of major epicardial marker gene *Wt1* and EMT‐related genes was not changed while epithelial genes expression decreased, indicating EpiSV40 might be more comparable to the primary epicardial cells on the EMT process (Figure S1B). Immunostaining of EdU, pH3 and Ki67 exhibited that both of the primary epicardial cells P1 and EpiSV40 responded uniformly to serum stimulation, though EpiSV40 had a higher basal proliferation level (Figure 5A‐D). Meanwhile, the EdU, pH3 or Ki67 positive cells can be barely detected in primary epicardial cells P3, indicating the loss of proliferation capacity after serial passage (Figure S1C).

**FIGURE 6:**
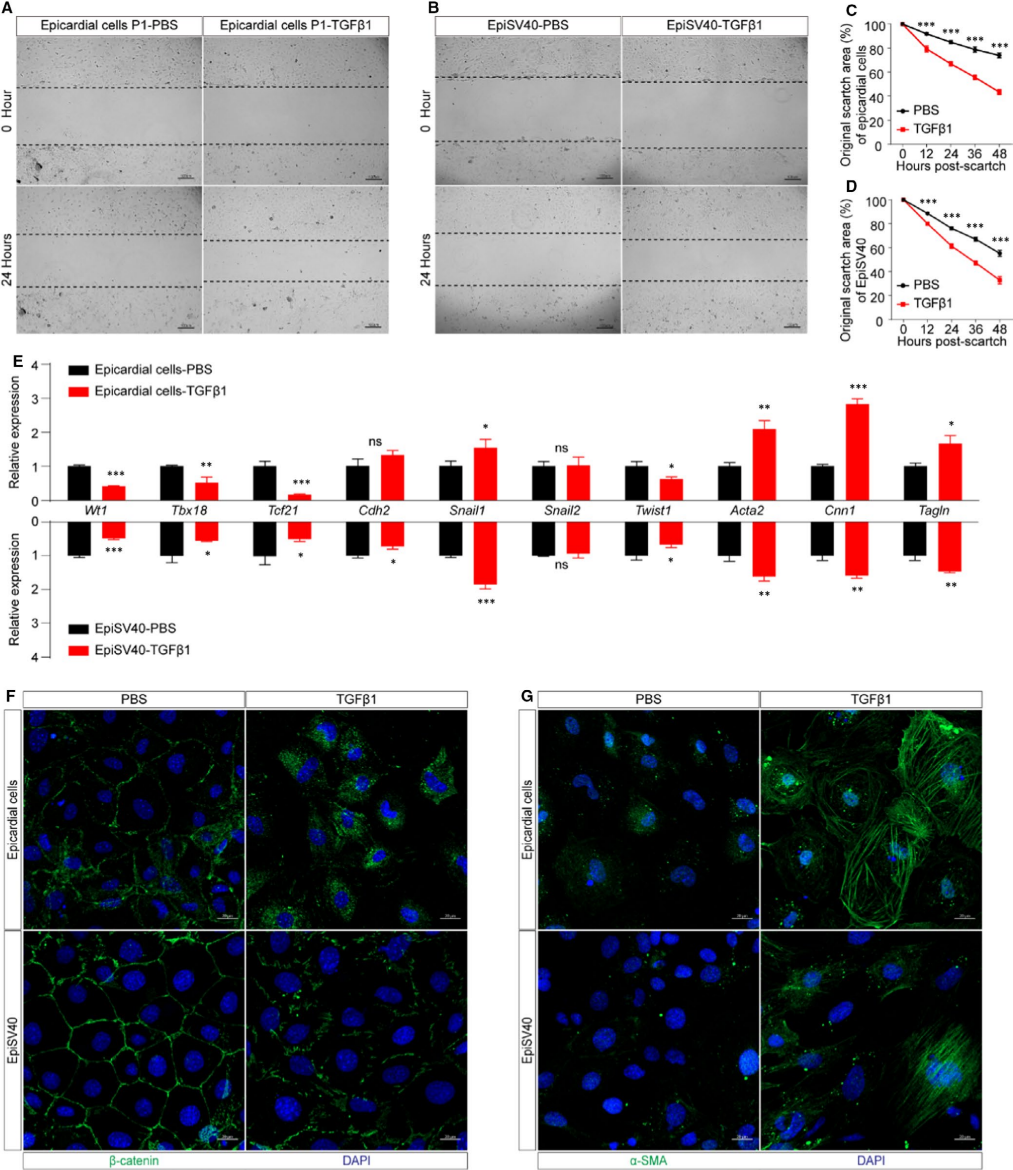
The migration and differentiation capacities of primary epicardial cells and EpiSV40. A, Representative images of cell migration of primary epicardial cells. Scale bar, 100 µm. B, Representative images of cell migration of EpiSV40. Scale bar, 100 µm. C, Migration of primary epicardial cells is presented as a percentage of the original scratch area (n = 6 per group). D, Migration of EpiSV40 is presented as a percentage of the original scratch area (n = 6 per group). E, Fold changes in gene expression (n = 4 per group). NS, not significant, ^∗^ *P* < 0.05, ^∗∗^ *P* < 0.01 and ^∗∗∗^ *P* < 0.001. F, Primary epicardial cells and EpiSV40 were stained with β‐catenin (green) and DAPI (blue). Scale bar, 20 µm. G, Primary epicardial cells and EpiSV40 were stained with α‐smooth muscle actin (α‐SMA) (green) and DAPI (blue). Scale bar, 20 µm

TGFβ1 has been widely used to examine the capacity of migration and differentiation of the primary epicardial cells.^30^, ^31^, ^32^ Scratch wound healing assay revealed that EpiSV40 migration was enhanced significantly by TGFβ1, which coincides with primary epicardial cells (Figure 6A‐D). Immunostaining showed that EpiSV40 differentiation was induced by TGFβ1 with β‐catenin decrease and up‐regulation of α‐SMA, a marker of the vascular smooth muscle cell (VSMC) (Figure 6F‐G). We detected the expression of EMT and VSMC‐related genes by qRT‐PCR and found that EpiSV40 has a similar response to TGFβ1 stimulation compared to the primary epicardial cells (Figure 6E), indicating EpiSV40 is suitable for EMT and VSMC differentiation research.

In conclusion, EpiSV40 had comparable proliferation, migration and differentiation capacities as primary epicardial cells, suggesting it is an adapted tool for epicardial cell in vitro study.

## 4 DISCUSSION

The epicardium plays an important role in cardiac development and heart repair.^3^, ^4^, ^5^, ^6^, ^7^, ^8^ Here, we established and characterized an immortalized mouse epicardial cell line EpiSV40, which maintained the morphology and proliferation, migration and differentiation abilities of primary epicardial cells, suggesting its utility as an ideal in vitro study model for epicardium research.

Primary culture of epicardial cells from the embryonic heart was a difficult process, and there are two methods for primary epicardial cells isolation: tissue attachment and FACS. The technical comparison of these two methods from different groups is detailed in Table 2. In brief, the tissue attachment method is gentler and has less impact on primary epicardial cells, in which cell morphology approximates the situation in vivo.^12^ The FACS method requires enzymatic digestion and flow cytometry, which has a great impact on the cells, and the sorted cells are spindle‐shaped mesenchymal cells.^3^ Besides, this method is more laborious with additional steps, such as the non‐specific labelling method of CMFDA incubation or the transgenic mice line carrying the fluorescent reporter for labelling the epicardium.^26^, ^33^, ^34^ We obtained highly purified primary epicardial cells with cobblestone morphology using the tissue attachment method, which showed a robust expression of epicardial markers.

**TABLE 2:** Differences in epicardial cell isolation and cell biology

References	Cell acquisition	Mice required	Gestational age of mice	Main components of culture medium	Coating medium	Cell morphology	Cell type	Cell proliferation	Cell migration	Cell differentiation	Cell passage	Cell immortality
Austin, A. F. et al, Dev Dyn, 2008^31^	Tissue attachment	Transgenic mice: Tg(H2‐K1‐tsA58)6Kio/LicrmJ	E10.5, E11.5 or E13.5	DMEM 10% FBS ITS MGI	Collagen	Cobblestoned	Epithelial	NA	NA	Yes	Yes	Yes
Zhou, B. et al, Methods Mol Biol, 2012 ^38^	Collagenase digestion + FACS	Transgenic mice: *Wt1^GFPCre^* *Wt1^CreERT2^* *Rosa26^mTmG^*	E14.5, E15.5 or adult	MSCGM 20% FBS	Gelatin	Spindled	Mesenchymal	NA	NA	Yes	Yes	No
Velecela, V. et al, Methods Mol Biol, 2016 ^36^	Tissue attachment	Transgenic mice: Tg(H2‐K1‐tsA58)6Kio/LicrmJ	E11.5	DMEM 10% FBS pyruvate	Gelatin	Cobblestoned	Epithelial	NA	Yes	NA	Yes	Yes
Trembley, M. A. et al, J Vis Exp, 2016 ^32^	Tissue attachment	Wild type	E11.5	M199 5% FBS	Collagen	Cobblestoned	Epithelial	NA	NA	Yes	NA	No
Ramesh, S. et al, J Vis Exp, 2016 y^12^	Tissue attachment	Wild type	E11.5 or E12.5	DMEM 10% FBS FGF2	NA	Cobblestoned	Epithelial	NA	Yes	Yes	NA	No
This study	Tissue attachment	Wild type	E11.5	DMEM 10% FBS	Gelatin	Cobblestoned	Epithelial	Yes	Yes	Yes	Yes	Yes

Abbreviations: FACS, Fluorescence‐activated Cell Sorting; FGF2, recombinant fibroblast growth factor 2; ITS, Insulin transferrin selenium; MGI, mouse gamma interferon; MSCGM, Mesenchymal stem cell growth medium; NA, not available.

As shown in our study, the major constrain of the primary epicardial cells in vitro culture is low cell number and limited life span. Immortalization of the primary mammalian cells is a simple and feasible approach to the manufacture of target cells for in vitro culture. *SV40‐LT* is the most widely used gene for cell immortalization by inhibiting p53 functions to prolong cell cycle.^35^ A large number of *SV40‐LT*‐mediated immortalized cell lines have been developed with comparable biological characteristics to primary cells.^20^, ^21^, ^22^, ^23^, ^24^, ^25^ In the present study, we successfully constructed an epicardial cell line (EpiSV40) by *SV40‐LT*‐mediated immortalization. Further analysis demonstrated that EpiSV40 has comparable proliferation, migration and differentiation capacities as primary epicardial cells. Transgenic mice (ImmortoMouse) have been used to generate immortal epicardial cells for cell differentiation research,^31^, ^36^ but developmental defects like thymic hyperplasia influence the survival of this mice line.^37^ The EpiSV40 preparation method we proposed is feasible without the requirement of genetically engineered mice.

To sum up, we established an easy culture epicardial cell line, EpiSV40, to substitute primary epicardial cells for mechanism research. EpiSV40 has the cobblestone morphology and comparable functions to primary cells. We believe the application of EpiSV40 will facilitate the study of the epicardium in cardiac development and heart repair.

## CONFLICT OF INTEREST

The authors declare no competing conflicts of interest.

## AUTHOR CONTRIBUTIONS

**Haobin Jiang:** Conceptualization (equal); Investigation (equal). **Shen Song:** Writing‐original draft (equal). **Jiacheng Li:** Methodology (equal). **Qianqian Yin:** Formal analysis (equal). **Shengshou Hu:** Funding acquisition (equal); Supervision (equal). **Yu Nie:** Supervision (equal); Writing‐original draft (equal); Writing‐review & editing (equal).

## Supporting information

Fig S1

Table S1

## Data Availability

The data that support the findings of this study are available from the corresponding author upon reasonable request.
