# Supplementary material for: Establishment and characterization of an immortalized epicardial cell line
Source: J Cell Mol Med. 2021 Apr 6;25(13):6070–81. doi: 10.1111/jcmm.16496 (PMC8406488; doi:10.1111/jcmm.16496)
Supplement: Supplementary file 1 — Fig S1 [file JCMM-25-6070-s002.docx]

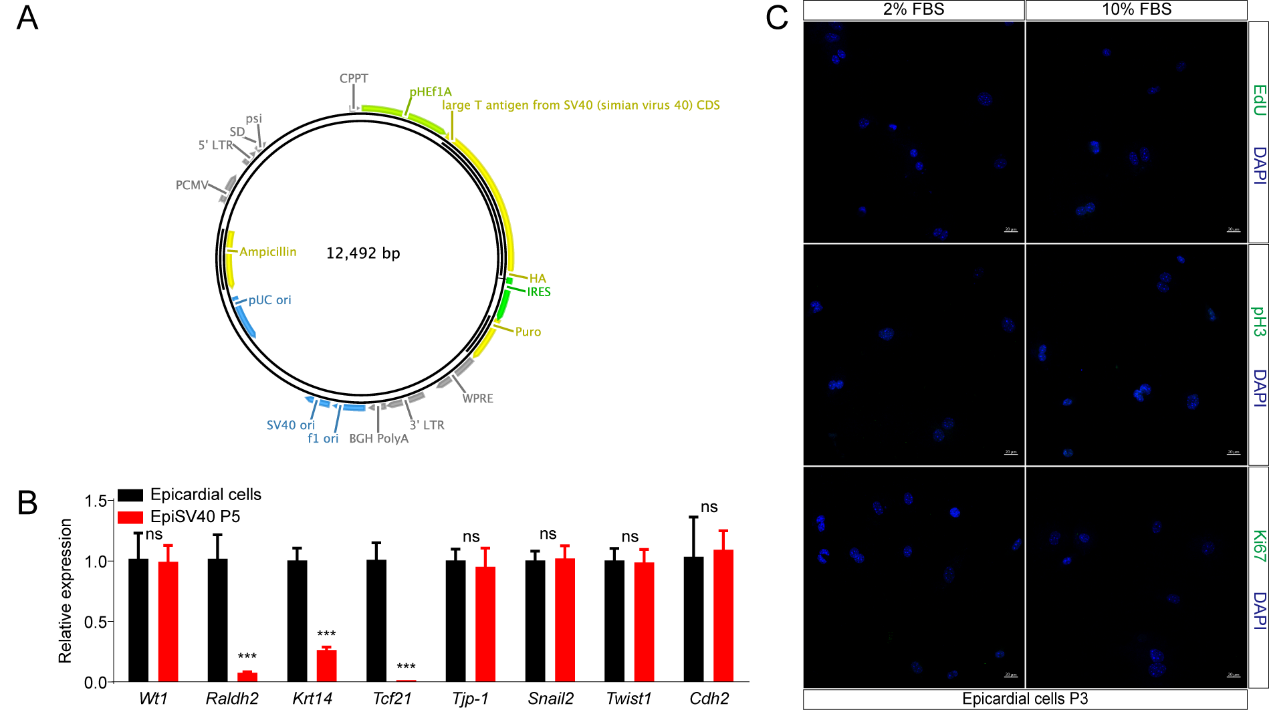


**Supplement Figure 1**

**A,** Schematic diagram of plasmid pLVX-IRES-Puro-SV40LT. **B,** Fold changes in gene expression (n=4 per group). NS, not significant, ∗p < 0.05, ∗∗p < 0.01, and ∗∗∗p < 0.001. **C,** Proliferating cells were detected via EdU (green), pH3 (green), Ki67 (green), and DAPI (blue). Scale bar, 25 µm.
